# The effects of microglia‐ and astrocyte‐derived factors on neurogenesis in health and disease

**DOI:** 10.1111/ejn.14969

**Published:** 2020-09-21

**Authors:** Tasuku Araki, Yuji Ikegaya, Ryuta Koyama

**Affiliations:** ^1^ Laboratory of Chemical Pharmacology Graduate School of Pharmaceutical Sciences The University of Tokyo Tokyo Japan; ^2^ Center for Information and Neural Networks Suita City Osaka Japan

**Keywords:** dentate gyrus, glia, granule cell, hippocampus

## Abstract

Hippocampal neurogenesis continues throughout life and has been suggested to play an essential role in maintaining spatial cognitive function under physiological conditions. An increasing amount of evidence has indicated that adult neurogenesis is tightly controlled by environmental conditions in the neurogenic niche, which consists of multiple types of cells including microglia and astrocytes. Microglia maintain the environment of neurogenic niche through their phagocytic capacity and interaction with neurons via fractalkine‐CX3CR1 signaling. In addition, microglia release growth factors such as brain‐derived neurotrophic factor (BDNF) and cytokines such as tumor necrosis factor (TNF)‐α to support the development of adult born neurons. Astrocytes also manipulate neurogenesis by releasing various soluble factors including adenosine triphosphate and lactate. Whereas, under pathological conditions such as Alzheimer's disease, depression, and epilepsy, microglia and astrocytes play a leading role in inflammation and are involved in attenuating the normal process of neurogenesis. The modulation of glial functions on neurogenesis in these brain diseases are attracting attention as a new therapeutic target. This review describes how these glial cells play a role in adult hippocampal neurogenesis in both health and disease, especially focusing glia‐derived factors.

## Graphical abstract

Microglia and astrocytes support adult neurogenesis in the healthy brain through physical contacts and release of soluble factors. In the diseased brain, the glial support functions are lost in some cases and adult neurogenesis is impaired by glia‐derived inflammatory signals. Thus, recovery of adult neurogenesis by regulating glial functions could be new therapeutic strategies in adult neurogenesis‐related brain diseases such as epilepsy and depression.

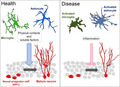

**Abbreviations**
abGC: adult‐born granule cell
AD: Alzheimer's disease
APP: amyloid precursor protein
AQP4: aquaporin‐4
ATP: adenosine triphosphate
Aβ: amyloid β
BDNF: brain‐derived neurotrophic factor
BrdU: 5‐bromo‐2′‐deoxyuridine
CX43: connexin 43
DCX: doublecortin
E: embryonic
FGF‐2: fibroblast growth factor 2
FGFR1: fibroblast growth factor receptor 1
GABA: gamma‐amino butyric acid
GDNF: glial cell line‐derived neurotrophic factor
GFAP: glial fibrillary acidic protein
IFN: interferon
IGF‐I: insulin‐like growth factor I
IGFIR: insulin‐like growth factor I receptor
IL: interleukin
IPC: intermediate progenitor cell
LPS: lipopolysaccharide
MCT: monocarboxylate transporter
MHC: major histocompatibility complex
NO: nitric oxide
NPC: neural progenitor cells
NSC: neural stem cells
P: postnatal
PEPCK‐M: mitochondrial phosphoenolpyruvate carboxykinase
PIK3R2: phosphoinositide‐3‐kinase regulatory subunit 2
PS: phosphatidylserine
PS1: human presenilin‐1
PSA‐NCAM: polysialic acid attached neural cell adhesion molecule
SCID: severe combined immunodeficiency
SE: status epilepticus
SGZ: subgranular zone
SNRI: Serotonin Noradrenaline Reuptake Inhibitors
Sox2: SRY‐box 2
SSRI: selective serotonin reuptake inhibitor
SVZ: subventricular zone
Tbr2: T‐box brain protein 2
TLR: Toll‐like receptor
TNF: tumor necrosis factor
TNFRI: tumor necrosis factor‐α receptor I
VEGF: vascular endothelial growth factor
VPS35: Vacuolar sorting protein 35

## 1 INTRODUCTION

During brain development, neural stem cells (NSCs) actively proliferate and differentiate into neurons; these activities are rapidly followed by the development of functional neural circuits. Using tritiated thymidine to trace dividing cells in rats and guinea pigs, Altman became the first to suggest that neurons could be newly generated in adulthood (Altman & Das, 1965, 1967). In the 1990s, evidence of adult neurogenesis was also reported in other mammals including rodents, monkeys, and humans (Eriksson et al., 1998; Kornack & Rakic, 1999; Kuhn et al., 1996). At this time, neurogenesis research came into the spotlight. To date, an increasing number of studies have demonstrated that NSCs are present in two regions in the mammalian adult brain, namely, the subventricular zone (SVZ) and the subgranular zone (SGZ) of the dentate gyrus in the hippocampus, and that new neurons continue to be produced in these regions (Gage, 2000).

**FIGURE 1:**
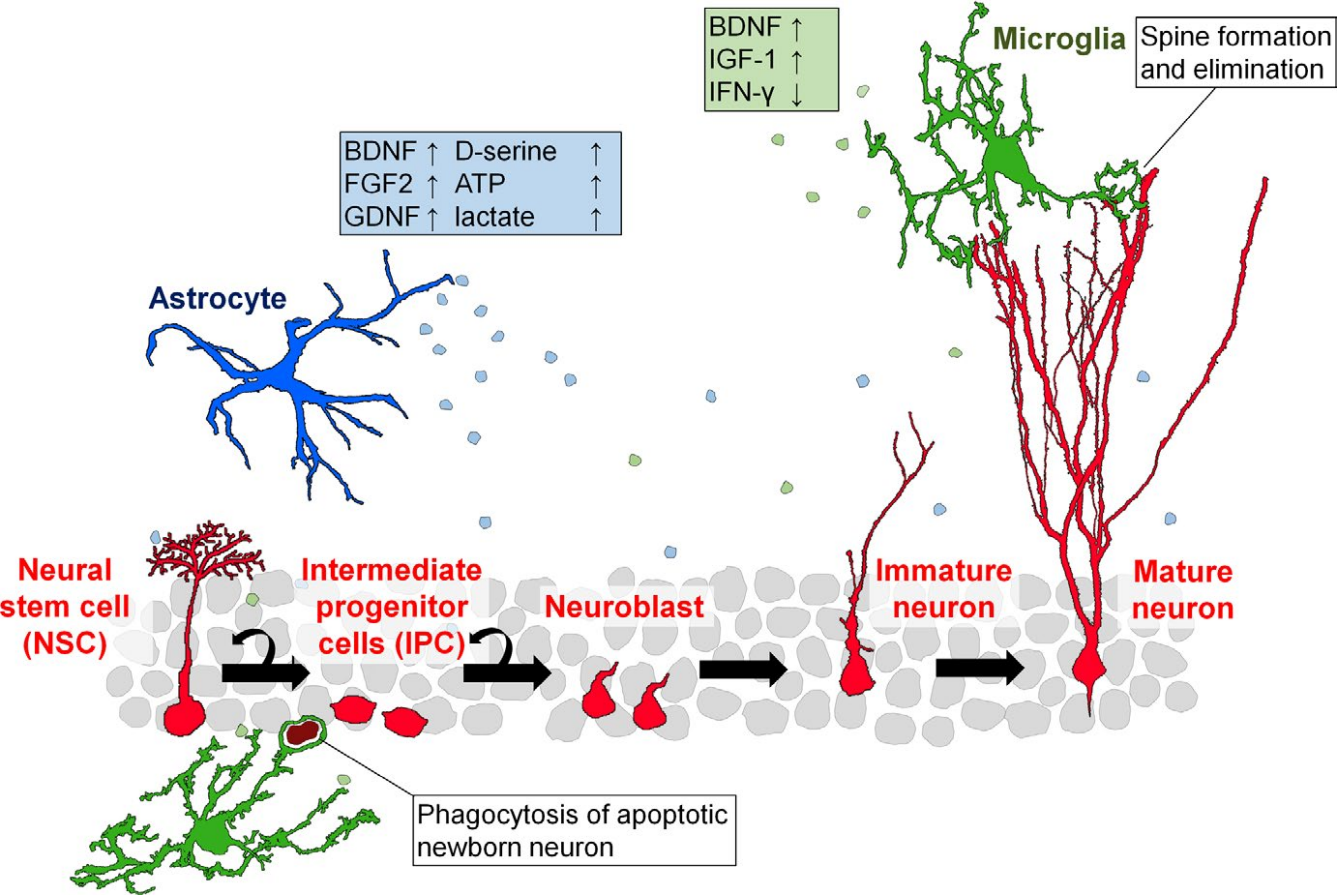
Glial support of hippocampal neurogenesis in health. Neural stem cells (NSCs) in the hippocampal subgranular zone produce intermediate progenitor cells (IPCs) through asymmetric division. Furthermore, IPCs differentiate into neuroblasts while continuing to proliferate, and grow into mature hippocampal granule cells. Microglia and astrocytes support these processes. Soluble factors released from microglia, such as brain‐derived neurotrophic factor (BDNF) and insulin‐like growth factor I (IGF‐1), and astrocytic fibroblast growth factor 2 (FGF‐2) and D‐serine, promote processes such as proliferation, differentiation, and development of NSCs and IPCs. In addition, microglia induce spine formation through physical contact with their processes and phagocytose synapses and apoptotic adult‐born granule cells to support neurogenesis. ATP, adenosine triphosphate; IFN‐γ, interferon‐γ

In this review, we focused on the role of microglia and astrocytes in the process of neurogenesis in SGZ of rodents. Glial cells are a major component of the neurogenic niche, and how they support neurogenesis is of great interest (Figure 1). Microglia, the brain‐resident immune cells, monitor and survey the surrounding environment by constantly extending and contracting their processes (Nimmerjahn et al., 2005). It is shown that functions such as physical contact between microglia and neurons and the release of soluble factors by microglia are beneficial for various stages of neurogenesis (Sato, 2015). It has also been suggested that the promotion of neurogenesis by voluntary exercise or enriched environment requires microglial support (Kohman et al., 2012; Xu et al., 2016). Astrocytes have the same origin as neurons, differentiating from NSCs. The similarity of genes expressed by astrocytes and NSCs has made it difficult to investigate the specific role of astrocytes in neurogenesis (Cassé et al., 2018). However, an increasing number of studies have suggested that astrocytes can control the growth and differentiation of adult‐born granule cells (abGCs) through the release of various trophic factors and gliotransmitters (Quesseveur et al., 2013; Sultan et al., 2015). They may also supply energy, such as lactate, that is essential for the growth of abGCs (Alvarez et al., 2016).

In pathological conditions, however, glial cells can be a major cause of abnormal neurogenesis. They are known to both mediate the inflammatory response and impair neurological function in neurodegenerative diseases (Ekdahl et al., 2003; Iram et al., 2016). We will review the role of glial cells on neurogenesis in Alzheimer's disease (AD), a neurodegenerative disease with decreased neurogenesis; depression, a psychiatric disorder with decreased neurogenesis; and epilepsy which is often accompanied with transient overgrowth of neurogenesis and subsequent depletion. We will summarize research findings that show how glial cells are involved in neurogenesis in both health and disease.

## 2 ADULT HIPPOCAMPAL NEUROGENESIS

In mouse, development of the dentate gyrus is mostly complete by the first two postnatal weeks, but production of new granule cells continues throughout life (Muramatsu et al., 2007; Nicola et al., 2015). It was reported that each day, one abGC is created for every 2,000 existing granule cells in 2 months old mice (Kempermann et al., 1997). Compared with granule cells born during development, abGCs have more dendritic spines and larger axonal boutons, suggesting that abGCs have unique functions and roles (Cole et al., 2020). Behavioral analyses have shown that abGCs are required for functions such as hippocampal‐dependent spatial learning (Drapeau et al., 2003; Kempermann & Gage, 2002), fear conditioning (Drew et al., 2010; Saxe et al., 2006), and pattern separation (Clelland et al., 2009; Sahay et al., 2011).

The process of adult hippocampal neurogenesis from NSC to mature neuron is classified into multiple stages depending on the expression of molecular markers. NSCs, also known as radial glial‐like cells, express markers such as nestin, glial fibrillary acidic protein (GFAP), and SRY‐box 2 (Bonaguidi et al., 2011). NSCs can differentiate into both neurons and astrocytes. Although many NSCs are quiescent, they are capable of self‐renewal and asymmetric division to give rise to intermediate progenitor cells (IPCs; Bonaguidi et al., 2011). IPCs are primarily identified by the expression of T‐box brain protein 2 and become neuroblasts with a limited number of divisions (Hodge et al., 2008; Lugert et al., 2012). Neuroblasts that express doublecortin (DCX) migrate into the granule cell layer and ultimately differentiate into mature neurons (Berg et al., 2015; Kempermann et al., 2004). However, it should be noted that the above molecular markers can be expressed in multiple stages in the process of neurogenesis. For example, nestin was expressed in early‐phase IPCs as well as NSCs, and DCX is expressed in late‐phase IPCs (Hussaini et al., 2013).

It should be noted that the expression marker‐dependent classification of neurogenic phase remains vague in some studies. For example, 5‐bromo‐2′‐deoxyuridine (BrdU), a frequently‐used marker for dividing cells, labels dividing cells in S‐phase. Thus, it should be carefully taken into consideration that depending on the BrdU labeling paradigm used, BrdU addresses different biological questions and this is why BrdU labeling alone cannot determine the specific stage of neurogenesis. Furthermore, in SGZ, several cell types are proliferating, which allows BrdU‐labeling of these cells: IPCs are transient and highly proliferative; NSCs are largely quiescent but recruited under some conditions; early neuroblasts transitioning from IPCs retain some proliferation. The field of neurogenesis suffers an excess of “nomenclaturing” and each cell type has several names: NSCs are also called radial glia, radial neural progenitor cell (NPCs), and quiescent neural progenitor cells (QNPs), or type I cells. Their daughter cells have been called transient amplifying progenitor cells, amplifying neuroprogenitors, NPCs (as they are already committed to the neuronal lineage), or type II cells (types IIa and IIb); neuroblasts are also called type III cells. In the current review, we basically used each term as it appeared in the original references.

For the differentiation of NPCs and their incorporation into existing neural circuits, proper formation of a neurogenic niche, which is comprised of multiple cell types and the extracellular matrix, is important: it has been suggested that various factors including glial cells, such as astrocytes and microglia, blood vessels, extracellular matrix, and neural activity create a microenvironment suitable for neurogenesis (Artegiani et al., 2017). Indeed, adult rat spinal cord NPCs differentiated into glial cells when transplanted into CA1 but differentiated into neurons when transplanted into the dentate gyrus (Shihabuddin et al., 2000), suggesting that the neurogenic niche in the dentate gyrus, i.e., the SGZ, is able to support and enhance neurogenesis.

Despite the clear evidence of adult neurogenesis in rodents, human adult neurogenesis remains a controversial topic. The studies that utilized BrdU labeling claimed a lifelong neurogenesis in human SGZ because BrdU‐labeled cells were found in SGZ of elderly individuals (Boldrini et al., 2009, 2018; Eriksson et al., 1998). However, Sorrells and colleagues have shown that human neurogenesis in the SGZ declines sharply after the first year of life and is very low at ages 7 and 13 (Sorrells et al., 2018). In addition, the authors concluded that neurogenesis in the dentate gyrus does not continue or is very rare in adults because few immature neurons (DCX‐ and PSA‐NCAM (polysialic acid attached neural cell adhesion molecule)‐expressing neurons) were found in epilepsy patients or adult monkeys (Sorrells et al., 2018). These seemingly inconsistent findings may result from the conditions of fixation, storage, and staining of post‐mortem brain tissues. Moreno‐Jiménez and colleagues have examined the conditions of tissue processing in detail, finding that thousands of immature neurons were observed even in the hippocampus of people over 90 years old (Moreno‐Jiménez et al., 2019). Due to the limitations of the use of live brain tissues in human research, the field of human neurogenesis research requires the development of non‐invasive research methods (Kumar et al., 2019).

## 3 MICROGLIA IN ADULT NEUROGENESIS

Microglia account for 5%–10% of the cells in the brain and serve as immune cells in the central nervous system. They are distinct from other brain cells in that they originate from the mesoderm, while neurons and astrocytes originate from the ectoderm (Marín‐Teva et al., 2012). Fate mapping analysis revealed that immature macrophages generated in the yolk sac enter the central nervous system when the circulatory system begins to form (around embryonic day 8.5 [E8.5]; Ginhoux et al., 2010). The immature macrophages that enter the brain proliferate and are differentiated to microglia.

In the healthy brain, microglia exist in a ramified form with branched processes around the soma; they expand and contract their processes to monitor the surrounding brain environment (Nimmerjahn et al., 2005). Microglia contribute to the maintenance of homeostasis by interacting with surrounding brain cells, and supporting their structure and function by both direct physical contact and the release of soluble factors (Miyamoto et al., 2016; Parkhurst et al., 2013). In addition, microglia prune synapses by phagocytosis and refine neural circuits during development (Paolicelli et al., 2011; Schafer et al., 2012).

It has been reported that microglia also contribute to the regulation of adult neurogenesis. Genetic removal of microglia reduced the survival of neuroblasts in the adult hippocampus (Kreisel et al., 2019). It has also been shown that microglia in the neurogenic niche exhibit a different heterogeneity compared with microglia in other brain areas. For example, microglia expressing the phagocytosis associated gene, *Clec7a*, are present in limited neurogenic regions such as SVZ and SGZ (Stratoulias et al., 2019). Microglia in the dentate gyrus alter transcription levels and phenotypes in response to neurogenesis‐promoting factors such as vascular endothelial growth factor (VEGF; Kreisel et al., 2019). These findings suggest that microglial functions in the neurogenic niche are spatiotemporally modulated to maintain the homeostasis of neurogenic regions in the brain. Several of the roles microglia play in neurogenesis are introduced below.

### 3.1 Phagocytosis

More than half of abGCs in the SGZ die through apoptosis within the first few weeks of being generated (Dayer et al., 2003). If apoptotic cells are not removed, they may scatter organelles and other cellular components that could cause harmful inflammation in the surrounding environment. It has been shown that apoptotic abGCs are quickly recognized and removed by microglia and possibly other cells, including NPCs (Lu et al., 2011; Sierra et al., 2010). It should be also noted that the engulfment of dead abGCs is carried out by ramified microglia without obvious inflammation (Sierra et al., 2010); microglia that are not exhibiting fat and short processes phagocytose dead abGCs by wrapping the ends of their branched processes around the dead cell, i.e., formation of the phagocytic cup.

Although there are many unclear points about the molecular mechanisms underlying the removal of apoptotic abGCs by microglia, the involvement of several molecules has been proposed. Phosphatidylserine (PS), a phospholipid that normally found only inside the lipid bilayer, is exposed on the surface of apoptotic cells and acts as an ‘eat me’ signal for engulfment by phagocytic cells (Elmore, 2007; Nagata et al., 2010). The expression of PS receptors in the brain remains controversial; however, it has been reported that microglia express them for the phagocytosis of apoptotic cells (De Simone et al., 2002; Witting et al., 2000). Furthermore, intravenous administration of annexin V, which binds to the PS receptor and inhibits PS recognition, caused accumulation of apoptotic cells in DG and reduced the viability of immature neurons without affecting the number of BrdU‐labeled proliferating cells (Lu et al., 2011). These results suggest that the importance of elimination of dead abGC via PS recognition to maintain the homeostasis of neurogenic niche.

In addition, the complement protein C1q contributes to the removal of apoptotic abGC (Diaz‐Aparicio & Sierra, 2019). C1q mRNA and protein were mainly produced by microglia in the hippocampus under physiological conditions. In the dentate gyrus, the majority of apoptotic cells that were phagocytosed by microglia were surrounded by C1q‐localized microglial processes. Since C1q binds to PS and opsonizes apoptotic cell (Païdassi et al., 2008), these proteins likely support the rapid clearance of dead abGC.

It has been suggested that phagocytic microglia had elevated expression of genes that encode trophic factors and peptides associated with both positive and negative regulation of neurogenesis (Diaz‐Aparicio et al., 2020). Furthermore, conditioned medium from phagocytic microglia suppressed the differentiation of NSCs into neural cells, suggesting that phagocytic microglia contribute to the maintenance of the NSC pool. Indeed, highly‐promoted neurogenesis can cause disadvantages such as depletion of NSCs and early cessation of neurogenesis, as well as benefits such as enhancing hippocampus‐dependent behaviors in experimental animals (Scharfman & Hen, 2007; Sierra et al., 2015).

In the regulation of neurogenesis, microglial phagocytosis may target not only apoptotic cells but also synapses and extracellular matrix. New neurons extend axons and dendrites to form synapses and integrate into existing circuits. However, multiple types of cells, synapses, and extracellular matrix already fill the space in the target region, which may hinder the synapse formation of abGC. Thus, abGCs should find or create space generating synapses in existing circuits.

Synaptic competition may be one of the mechanisms by which abGCs integrate into existing neural circuits effectively. abGCs extend axons toward CA3 pyramidal cells and compete with mature granule cell axons to form synapses on the dendrites of pyramidal cells in a neuronal activity‐dependent manner (Yasuda et al., 2011). It has been suggested that the synapses formed by abGC are incorporated into neural circuits to replace old synapses (Toni et al., 2007), and old synapses may be removed by microglia. During development, neuronal activity‐dependent clearance of inactive synapses by microglia is an essential process in the formation of healthy neural circuits (Schafer et al., 2012). Thus, it is possible that synaptic engulfment by microglia is involved in synaptic clearance during the integration of abGCs into existing circuits in the adult brain. Whether old synapses that have lost in competition with abGC will be eliminated by microglia needs to be further investigated. However, in a mouse model of autism using maternal immune activation, excess synapses in CA3 were removed by microglial engulfment which was induced by voluntary exercise‐induced synaptic competition in the hippocampus, demonstrating the ability of microglia to remove synapses in the adult brain (Andoh et al., 2019). In addition, microglia have been shown to reconstitute hippocampal synapses by trogocytosis (Weinhard et al., 2018). Therefore, it will be interesting to test the idea that microglia help the integration of abGC by removing old synapses.

### 3.2 Secretion of soluble factors

#### 3.2.1 Brain‐derived neurotrophic factor

Microglia can secrete various soluble factors, including trophic factors and cytokines that can control neurogenesis (Elkabes et al., 1996; Kim & De Vellis, 2005). Brain‐derived neurotrophic factor (BDNF) and insulin‐like growth factor (IGF)‐I are among the growth factors that have been shown to be secreted by microglia (Nakajima et al., 2001; Suh et al., 2013). The expression of these factors is decreased immediately after birth, but they continue to be expressed in adulthood in neurogenic brain regions such as the SVZ and the hippocampus (Dyer et al., 2016; García‐Segura et al., 1991; Mori et al., 2004).

Brain‐derived neurotrophic factor acts on abGCs and provides support for development at various stages of adult neurogenesis. Injection of BDNF into the rat hippocampus increased the number of BrdU+/NeuN + abGCs (Scharfman et al., 2005). Knockout of TrkB, a receptor for BDNF, from NPCs results in inhibition of their proliferation and a decrease in the thickness of the granule cell layer (Li et al., 2008). In addition, BDNF overexpression in mice increase the dendritic complexity of abGCs (Quesseveur et al., 2013). These results highlight the roles of BDNF in the proliferation, survival, and development of abGCs, but the origin of BDNF that causes these effects remains controversial. The role of BDNF in dendritic growth of abGCs has been shown to be mainly supported by autocrine BDNF production in experiments using abGC‐specific BDNF knockout mice (Wang et al., 2015). However, this result does not rule out the involvement of microglial BDNF in dendrite growth and spine formation. Studies using BDNF knockout mice in a CX3CR1 promoter‐dependent manner showed that microglial‐derived BDNF is required for learning‐induced dendritic spine formation in the motor cortex (Parkhurst et al., 2013). Interestingly, in this study, knockout of microglial BDNF does not affect total BDNF expression levels in the cortex and the hippocampus. Microglia may be able to regulate spine formation by locally extending their processes and release BDNF.

#### 3.2.2 Insulin‐like growth factor‐1

Insulin‐like growth factor‐1 is a growth factor expressed by microglia in the dentate gyrus during inflammation (Guthrie et al., 1995). It has been also reported that microglia express IGF‐1 without distinctive inflammation in human post‐mortem brain (Suh et al., 2013). IGF‐1 treatment induces NPCs proliferation in vivo and in vitro (Åberg et al., 2000; Åberg et al., 2003). One study with transgenic mice overexpressing IGF‐1 have reported that an increase in the number of dentate granule cells (O'Kusky et al., 2000) and another reported that the formation of the dentate gyrus was impaired in an NPC‐specific IGF‐I receptor knockout mouse (Liu et al., 2009). IGF‐1 positively regulates neurogenesis by promoting the NPC's proliferation, differentiation (Åberg et al., 2003; Yuan et al., 2015), and survival probably through exerting anti‐apoptotic effects (Liu et al., 2009).

Spontaneous exercise by the running wheel promotes NPC proliferation and survival, and interestingly, this effect may be mediated by microglial BDNF and IGF‐1 (Kohman et al., 2012; Littlefield et al., 2015). The voluntary exercise by wheel running increases the proportion of microglia that express BDNF and IGF‐1 in the dentate gyrus (Kohman et al., 2012; Littlefield et al., 2015). Studies using neurosphere cultures have shown that microglia are required for enhanced neurogenesis resulting from spontaneous exercise (Vukovic et al., 2012). In this study, hippocampal cell suspensions from mice that underwent 2‐week wheel running increased neurosphere formation, while this effect was abolished when microglia were selectively removed from cell suspensions. These results suggest that exercise induces a neuroprotective phenotype of microglia and promotes neurogenesis via the release of soluble factors.

#### 3.2.3 Tumor necrosis factor‐α

Factors such as brain disease, injury, and aging could induce different forms of activation in microglia. In response to changes in the surrounding environment, microglia change the expression patterns of various genes and become "activated microglia" that mediate inflammation (Keren‐Shaul et al., 2017; Masuda et al., 2019). In previous studies, lipopolysaccharide (LPS), a constituent of Gram‐negative bacterial cell wall outer membrane, has been widely used to experimentally investigate the influence of intracerebral inflammation on neurogenesis. Intraperitoneal or intracortical LPS administration did not affect the proliferation of NPCs, but inhibited their differentiation and survival (Ekdahl et al., 2003; Monje et al., 2003). Impairment of neurogenesis was positively correlated with microglial activation after LPS injection and minocycline, a tetracycline antibiotic which is often used to suppress microglial activation, treatment restored neurogenesis (Ekdahl et al., 2003). Since activated microglia release several inflammatory cytokines during inflammation, their effects on neurogenesis have been investigated.

Tumor necrosis factor (TNF)‐α is an inflammatory molecule that is released from microglia during inflammation (Welser‐Alves & Milner, 2013). TNF‐α secreted from microglia has been shown to induce apoptosis in hippocampal NPCs in vitro (Cacci et al., 2005). In epilepsy model mice, microglia release TNF‐α in a Toll‐like receptor 9 (TLR9)‐dependent manner and suppress abnormal increase in neurogenesis (Matsuda et al., 2015). These findings indicate that microglial‐derived TNF‐α suppresses neurogenesis. However, high concentrations of TNF‐α treatment have been shown to induce apoptosis of cultured NPCs in SVZ, while low concentrations of TNF‐α promote NPC proliferation and differentiation (Bernardino et al., 2008), suggesting a complex role for TNF‐α in adult neurogenesis. These complex regulation of neurogenesis may result from different receptor subtypes bound by TNF‐α. TNF‐α receptors‐1 (TNFR1) and ‐2 (TNFR2) were shown to be expressed in proliferating NPCs (Chen & Palmer, 2013). They reported that neurogenesis is enhanced in TNFR1 knockout mice and suppressed in TNFR2 knockout mice. In addition, TNFR2 and TNFα knockout mice had significantly reduced abGCs when radiation‐induced inflammation was induced. These results suggest that intracellular signaling via TNFR1 suppresses neurogenesis, while TNFR2 has a promoting effect. The role of TNF‐α has been focused mainly on the early steps in adult neurogenesis such as proliferation and survival. Because microglial TNF‐α can regulate excitatory synaptic connections by reducing CA1 pyramidal cell dendrites in hippocampal slice cultures (Liu et al., 2017), it is possible that microglial TNF‐α contributes to the synapse formation of abGCs.

#### 3.2.4 Interferon‐γ

Interferon (IFN)‐γ is also an inflammatory mediator that is released by microglia (Kawanokuchi et al., 2006; Mäkelä et al., 2010). Treatment of IFN‐γ reduced NPC proliferation and survival in vitro (Mäkelä et al., 2010). Similar results were reported when NPCs were treated with conditional medium of LPS‐treated microglia, and this effect was attenuated by neutralization with IFN‐γ antibody (Mäkelä et al., 2010). Consist with these results, the number of Ki‐67/PSA‐NCAM double‐positive NPCs were significantly increased, and the dendrites of dentate granule cells were enlarged in IFN‐γ knockout mice (Monteiro et al., 2015). In addition, hippocampal‐dependent spatial learning and recognition memory are enhanced in IFN‐γ knockout mice. These results suggest that IFN‐γ may negatively regulate proliferation, survival, and development of abGCs. Increased adult neurogenesis was reported in transgenic mice expressing a limited amount of IFN‐γ that does not cause inflammation or tissue abnormalities (Baron et al., 2008). The transgenic mice outperform the wild‐type mice in spatial learning and memory cognitive tests.

#### 3.2.5 Interleukins

It has been shown that virus‐mediated persistent overexpression of interleukin (IL)‐1β in the hippocampus impaired neurogenesis (Wu et al., 2012) and that injection of IL‐1β into the ventricle reduced the number of BrdU‐positive cells in the SGZ (Ja & Duman, 2008). Activated microglia immunostimulated by LPS and cytokines release a variety of factors that could affect neurogenesis, these include nitric oxide (NO) and other cytokines such as IL‐1β, IL‐6, and IL‐18 (Boje & Arora, 1992; Monje et al., 2003; Prinz & Hanisch, 1999; Sébire et al., 1993). Pharmacological and genetic inhibition of NO synthase increases cell proliferation in the neurogenic zone of the adult brain (Packer et al., 2003). Microglial‐derived IL‐1β stop cell cycle and induces apoptosis in NPCs under inflammation (Guadagno et al., 2015). In aged rats, microglial IL‐1β was associated with impaired neurogenesis (Gemma et al., 2007). Microglial conditioned medium stimulated with LPS reduced the number of neural progenitor cells, and the addition of IL‐6 antibody completely restored neurogenesis (Monje et al., 2003). IL‐18 also had a role in preventing the differentiation of NPCs into neurons (Liu et al., 2005). Thus, even though the involvement of microglia as a direct source of inflammatory mediators should continue to be carefully studied, current data suggest that microglia may negatively regulate neurogenesis by releasing inflammatory factors.

It has also been reported that the decreased neurogenesis and impaired memory that were observed following infection with West Nile virus were due to the persistent secretion of IL‐1β by activated astrocytes in the infected mice (Garber et al., 2018). However, repeated administration of IL‐6 and IL‐1β to the hippocampus significantly increased the number of abGCs (Seguin et al., 2009).

The conflicting findings of both positive and negative regulation of neurogenesis by IL6 and IL‐1β may be partly due to the differences in the concentration of cytokines used and the route of administration. Indeed, IL‐6 and IL‐1β increased neurogenesis when repeatedly administered into the hippocampus but did not affect neurogenesis when administered intraperitoneally (Seguin et al., 2009). Therefore, the roles of these cytokines under physiological conditions needs to be carefully evaluated.

### 3.3 Fractalkine‐CX3CR1 signaling

Fractalkine‐CX3CR1 signaling is an essential signaling pathway that regulates interactions between microglia and neurons (Harrison et al., 1998; Paolicelli et al., 2011). The ligand, fractalkine (also known as CX3CL1), is mainly expressed by neurons, including those in the hippocampus and dentate gyrus (Sheridan et al., 2014), whereas the fractalkine receptor, CX3CR1, is exclusively expressed by microglia in the brain parenchyma. Several lines of evidence have indicated that fractalkine‐CX3CR1 signaling is involved in adult neurogenesis. In CX3CR1 knockout mice, DCX‐positive cells and BrdU‐labeled cells were reduced compared to wild‐type mice, suggesting suppressed neurogenesis in these mice (Bachstetter et al., 2011). It was also shown that dendritic spines were reduced and synaptic vesicles were depleted in abGCs, and that abGCs were not properly integrated into the existing neural circuits in CX3CR1 knockout mice (Bolós et al., 2018). Deposition of extracellular matrix components such as lectin and aggrecan were confirmed in the molecular layer of the dentate gyrus; disruption of the environment surrounding the dentate gyrus may have affected the development of abGCs. Indeed, while extracellular matrix around dendrites suppresses spine remodeling (Orlando et al., 2012), microglia phagocytose extracellular matrix and help abGC spine formation (Nguyen et al., 2020).

Abnormalities of abGC dendrites in CX3CR1‐deficient mouse may be also due to impaired normal interactions between microglia and abGC. In vivo two‐photon time‐lapse imaging has demonstrated that spinal dynamics of newborn neurons in SVZ are reduced in CX3CR1‐deficient mice (Reshef et al., 2017). In this research, the contact between Cx3cr1‐deficient microglia and the dendrite shaft was reduced. The detailed regulatory mechanism remains unknown, but dendritic spines are regulated by local signal transduction via contact between microglia and dendrites, and this effect may also be effective in SGZ.

Dysfunction in CX3CR1‐mediated signaling may contribute to reduced neurogenesis in aged animals. Bachstetter et al. (2011) reported that fractalkine expression was decreased in aged wild‐type rats in association with decreased neurogenesis (Bachstetter et al., 2011). Administration of exogenous fractalkine to aged wild‐type rats stabilized the microglial morphology and promoted the proliferation of NPCs. Contrary with these findings, Sellner et al. (2016) reported that knockout of fractalkine did not affect the proliferation and survival of abGCs (Sellner et al., 2016). It suggests that the interaction between microglia and abGCs can be modulated by CX3CR1 ligands other than fractalkine, or that there are alternative pathways to fractalkine‐CX3CR1 signaling.

### 3.4 Communication with T cells

Some findings indicate that peripheral immune cells also play a role in regulating neurogenesis. Ziv et al. (2006) found that the number of abGCs was significantly decreased in both a mouse model of severe combined immunodeficiency (SCID), which lacks peripheral immune cells, and in nude mice lacking T cells (Kipnis et al., 2004; Ziv et al., 2006). These immunodeficient mice exhibited cognitive dysfunction, which was recovered by the recruitment of immune cells. T cells can be classified as either CD4‐positive or CD8‐positive cells. Helper T cells, which are CD4‐positive, are important for controlling neurogenesis (Wolf et al., 2009; Zarif et al., 2018). Helper T cells release IL‐4 and low concentrations of IFN‐γ and are able to induce microglial expression of IGF‐1 in vitro (Butovsky et al., 2006). Because IGF‐1 is known to promote neurogenesis, it is possible that interactions between T cells and microglia (via cytokine release) modulate neurogenesis, though T cells are severely restricted from entering the brain parenchyma by the blood‐brain barrier (Bechmann et al., 2007). In mice bred in an enriched environment, T cell infiltration to the dentate gyrus was observed and the expression of major histocompatibility complex‐II and IGF‐I in dentate gyrus microglia was increased (Ziv et al., 2006). It was shown that CD8‐positive, but not CD4‐positive T cells were necessary for the hippocampal neurogenesis and synaptic plasticity that was promoted by an enriched environment. The positive effects of an enriched environment on neurogenesis were abolished in the SCID mouse model (Butovsky et al., 2005). The contribution of T cell–microglia interactions to neurogenesis remains largely unknown and further studies are necessary to clarify the underlying mechanisms.

## 4 ASTROCYTES IN ADULT NEUROGENESIS

Astrocytes are the major cell type found in the neurogenic niche; they provide a special environment for adult neurogenesis. Astrocytes have long been recognized as "secretory cells" (Verkhratsky et al., 2016), mainly because they release, uptake, and store various factors that are essential for maintaining environmental homeostasis in the brain. Astrocytes also play an important role in synaptic transmission; they wrap around pre‐synapses and post‐synapses, forming a tripartite synapse, for the purpose of uptake or release of neurotransmitters and to control neuronal activity (Allen & Barres, 2009). In the mouse cortex, it is estimated that a single astrocyte makes contact with more than 100,000 synapses (Bunney et al., 2017), demonstrating the inseparable relationship between astrocytes and synaptic structure and function. An increasing number of studies have suggested that the aforementioned properties of astrocytes also play a role in promoting the differentiation and development of abGCs and controlling synapse integration into existing neural circuits. Additionally, the number of astrocytes is particularly high in the dentate gyrus compared to other brain areas (Olude et al., 2015), implying their active roles in controlling neurogenesis. We describe the evidence that astrocytes mainly regulate neurogenesis through the release of various factors below.

### 4.1 Secretion of growth factors

Song et al. (2002) first showed that astrocytes promote the differentiation of NSCs into neurons (Song et al., 2002). This effect was also achieved with coated substrates conditioned by primary astrocytes without direct contact between astrocytes and NSCs. In addition, astrocytes from the adult hippocampus showed stronger stimulation of neuronal differentiation than astrocytes from the adult spinal cord. This finding suggests that hippocampal astrocyte‐derived secretory factors promote neurogenesis. The astrocyte‐derived factors that may be promoting neurogenesis include BDNF, fibroblast growth factor 2 (FGF‐2), glial cell line‐derived neurotrophic factor (GDNF), and VEGF.

Precursor pro‐BDNF proteins secreted by neurons are captured by astrocytes through endocytosis; pro‐BDNF is then converted to mature BDNF (Bergami et al., 2008). Astrocytes are responsible for BDNF storage and could regulate BDNF levels in the brain (Rubio, 1997). Overexpression of BDNF in astrocytes promoted the survival and maturation of abGCs and induced anxiolytic‐/antidepressant‐like behaviors in mice (Quesseveur et al., 2013).

Fibroblast growth factor 2 also supports neurogenesis. A previous research has shown that when FGF‐2 was injected into the hippocampus of adult rats daily for 2 weeks, the number of DCX‐positive neuroblasts was increased and the dendritic maturation was enhanced (Rai et al., 2007). Fibroblast growth factor receptor 1 knockout mice showed the impairment of long‐term potentiation at the synapses between the medial perforant path and granule cells, exhibiting deficits in memory consolidation (Zhao et al., 2007).

In the SGZ of young adult mice, it was shown that about 90% of GFAP‐positive cells expressed FGF‐2 (Shetty et al., 2005). In the SVZ, GFAP‐positive cells were found to express FGF‐2 at high levels and they did not express nestin, a marker for NSCs, suggesting that the main source of FGF‐2 is astrocytes (Belluardo et al., 2008). In addition, the proportion of FGF‐2‐expressing astrocytes was dramatically reduced in correlation with age‐related decrease in neurogenesis (Shetty et al., 2005). These results suggest that astrocyte‐derived FGF‐2 supports abGC differentiation and functional integration.

Another example suggesting the role of astrocyte‐derived FGF‐2 in neurogenesis is the response to acute stress which was induced in rats after 3 hr of forced immobilization (Kirby et al., 2013). The authors showed that after exposure to acute stress, rats performed better in memory tests than controls who were not exposed to acute stress (Kirby et al., 2013). Acute stress increased FGF‐2 mRNA expression in the hilar astrocytes; blocking FGF‐2 inhibited the increase in neurogenesis that was observed after acute stress.

### 4.2 D‐serine

D‐serine is a neural modulator that is released from astrocytes under physiological conditions and acts as an agonist of the N‐methyl‐D‐aspartate receptor to induce long‐term potentiation of synaptic transmission in the hippocampus (Kang et al., 2013; Schell et al., 1995). Chronic intraperitoneal injection of D‐serine to adult mice for 8 days significantly increased the number of BrdU‐positive cells in SGZ (Sultan et al., 2013). In addition, D‐serine increased the number of DCX‐expressing cells and promoted the survival of abGCs. The phenomena may be mediated by an increased neuronal activity‐induced enhancement of synaptic integration of abGCs in the hippocampus (Panatier et al., 2006). A previous study showed that the inhibition of exocytosis in astrocytes resulted in attenuated dendritic formation in abGCs in association with reduced levels of extracellular D‐serine (Sultan et al., 2015). Interestingly, mosaic gene expression, which inhibits vesicle release in approximately half of astrocytes, resulted in the reduction of spine density only in the segment that interacted with the astrocytes in which vesicle release was inhibited. These results suggest that the regulation of dendritic spine formation by astrocyte‐derived D‐serine is extremely localized.

Astrocytic D‐serine‐mediated regulation of neurogenesis may also be involved in the pathogenesis of mental illness. In patients with schizophrenia, a decrease in D‐serine in both the cerebrospinal fluid and plasma and a decrease in neurogenesis have been confirmed (MacKay et al., 2019; Reif et al., 2006). Mice with astrocyte‐specific expression of a variant of a known schizophrenia risk gene, *Disrupted‐in‐Schizophrenia 1*, showed reduced neurogenesis and dysplasia of dendrites, which was restored by chronic administration of D‐serine (Terrillion et al., 2017). Thus, D‐serine is considered a potential treatment for schizophrenia (MacKay et al., 2019).

### 4.3 Adenosine triphosphate

As an energy source for cellular activity, adenosine triphosphate (ATP), is released as a purinergic neurotransmitter that mediates several functions in the brain (Cisneros‐Mejorado et al., 2014). Astrocytes can release ATP through exocytosis (Coco et al., 2003; Lalo et al., 2014). Vesicular secretion by exocytosis is triggered by increased intracellular Ca^2+^ ion concentrations via the inositol trisphosphate signaling pathway. Inhibition of phosphoinositide‐3‐kinase regulatory subunit 2 (PIK3R2) and inhibition of vesicle exocytosis by glycyl phenylalanine 2 naphthylamide reduced NPC proliferation in vitro (Cao et al., 2013). In PI3KR2 knockout mice, neurogenesis was dramatically reduced as assessed by the number of BrdU‐positive cells; administration of ATP into the ventricle rescued NPC proliferation (Cao et al., 2013). In addition, ectonucleotidase, an extracellular ATP hydrolase, is expressed exclusively in SVZ and SGZ (Lin et al., 2007). These results highlight the importance of ATP signals for neurogenesis. However, it is not clear whether ATP affects abGC development. For example, Ca^2+^ responses associated with purinergic signaling are restricted to NSC and NPC, and neuronal differentiation is accompanied with a loss of the Ca^2+^ responses (Lin et al., 2007). In addition, because microglia express various purinergic receptors such as P2X and P2Y that control their physiology, microglia are also target cells of ATP, which may result in manipulation of neurogenesis.

### 4.4 Lactate

Astrocytes store a large amount of glycogen, which is the energy source of cells and stored glycogen is degraded on demand, and astrocytes primarily supply energy to neurons (Brown & Ransom, 2007). Previous studies suggest that astrocytic glycogen is first broken down into lactate, released from the cell by monocarboxylate transporter (MCT) 4, and then taken up by neuronal MCT2 to be used as an energy source (Suzuki et al., 2011; Zhou et al., 2019). This pathway is known as the astrocyte–neuron lactate shuttle (Pellerin et al., 1998). Astrocytes have been confirmed to release lactic acid in vivo and in vitro (Sotelo‐Hitschfeld et al., 2015; Walz & Mukerji, 1988).

In the adult rodent brain, glucose is used as the main energy source. However, rats ≤3weeks of age show a relatively high expression of MCTs and a low expression of glucose transporters (Vannucci & Simpson, 2003), suggesting the importance of lactate as an energy source in the developing brain. Indeed, it has been shown that lactate, but not glucose, is necessary to maintain NPCs (Alvarez et al., 2016). Administration of the inhibitor of MCT or mitochondrial phosphoenolpyruvate carboxykinase, which is necessary for the metabolism of lactate, into the lateral ventricles of postnatal day 0 (P0) mice attenuated the maintenance of NPC self‐renewal (Alvarez et al., 2016). Additionally, it has been reported that the glycogen content is high in astrocytes in the dentate gyrus of young adult mice (Oe et al., 2016). This may be favored to supply of lactate to NPCs by the astrocyte‐neuron lactate shuttle. Thus, it is possible that the astrocyte‐NPC lactate shuttle supports neurogenic homeostasis.

However, it should be noted that lactate can be supplied to the brain through blood vessels. Lactate production by muscles is induced during intense exercise and the lactate concentration in blood significantly increases during and after exercise (Gladden, 2004). It has been demonstrated that increased lactate levels in the blood increase VEGF levels in the brain through binding of the hydroxycarboxylic acid receptor 1, which is expressed on vascular endothelial cells (Morland et al., 2017). Released VEGF promotes angiogenesis and neurogenesis (Jin et al., 2002; Pérez‐Escuredo et al., 2016; Zhou et al., 2018), suggesting that lactate‐mediated signaling is involved in exercise‐induced neurogenesis. It has been also shown that Intraperitoneal administration of lactate to mouse increase the viability of abGCs via MCT2 (Lev‐Vachnish et al., 2019).

These findings suggest that lactate modulates neurogenesis, but whether and how the astrocyte–neuron lactate shuttle affects neurogenesis needs to be further investigation, as the involvement of lactate in neurogenesis is only beginning to be elucidated.

### 4.5 Interleukins

Similar to microglia, astrocytes serve as mediators of inflammation and can release cytokines. Inflammation is considered harmful for neurogenesis, but some cytokines released may have neuroprotective functions. IL‐6 and IL‐1β are cytokines released from astrocytes (Iravani et al., 2012; Jones et al., 2018; Li et al., 2009) and they promote neuronal differentiation (Barkho et al., 2006). In IL‐6 knockout mice, NPC proliferation was reduced (Bowen et al., 2011) and hippocampus‐dependent learning was attenuated (Baier et al., 2009). Similar findings were also reported in astrocyte‐specific IL‐6 knockout mice (Erta et al., 2015). These results suggest that astrocytic IL‐6, produced under physiological conditions, promotes neurogenesis and supports cognitive function. However, contrary with these results, overexpression of astrocytic IL‐6 decreased neurogenesis and attenuated hippocampus‐dependent learning (Samuelsson et al., 2006; Valliéres et al., 2002).

## 5 NEUROGENESIS AND GLIA UNDER PATHOLOGICAL CONDITIONS

### 5.1 Alzheimer's disease

Alzheimer's disease is a neurodegenerative disease that accounts for 60 to 80% of the cases of dementia in elderly individuals (Barker et al., 2002). AD is characterized by impaired cognitive functions such as memory loss and language impairment. According to the amyloid β (Aβ) hypothesis, the underlying pathogenesis of AD is an accumulation of Aβ that results from abnormal cleavage of amyloid precursor protein (APP) by γ‐secretase, followed by abnormal phosphorylation and accumulation of tau (Hardy & Allsop, 1991; Hardy & Selkoe, 2002). However, it should be noted that the Aβ hypothesis is still influential, but there are some critical considerations (Kametani & Hasegawa, 2018).

**FIGURE 2:**
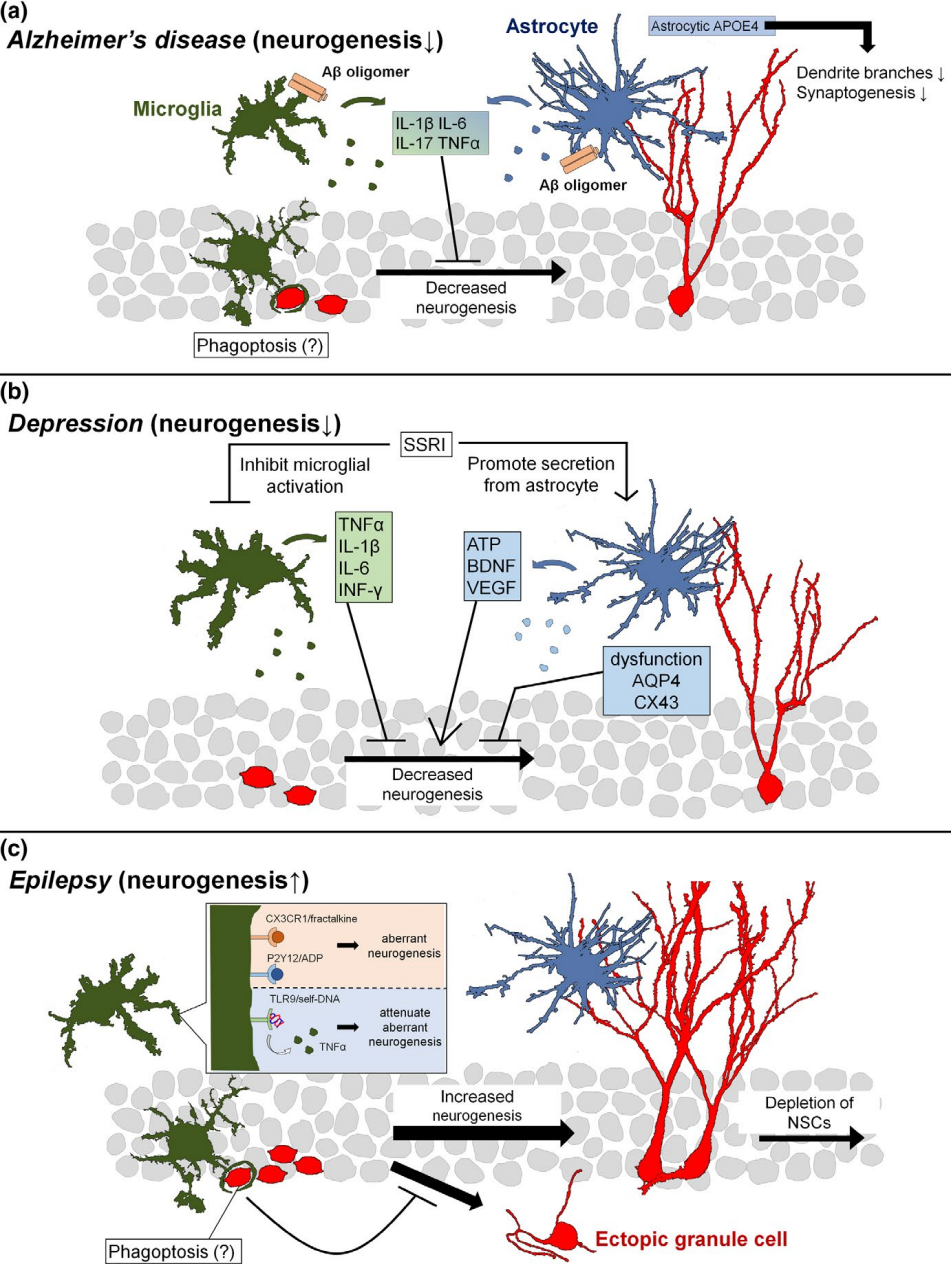
Glial modulation of hippocampal neurogenesis in disease. (a) In Alzheimer's disease, neurogenesis is decreased. When microglia and astrocytes are exposed to Aβ, they are activated and release cytokines that suppress neurogenesis, such as interleukin (IL)‐1β, IL‐6, IL‐17, and tumor necrosis factor (TNF)‐α. Expression of the Alzheimer's disease risk gene *APOE4* in astrocytes prevents synaptogenesis and induces reduced dendritic formation and shorter dendrites in newborn neurons. In addition, microglial phagoptosis may be involved in neural stem cell reduction. (b) Depression also shows reduced neurogenesis. Stress‐induced activation of microglia may result in the suppression of neurogenesis through the release of IL‐1β, IL‐6, TNF‐α, and interferon (IFN)‐γ. AQP4 and CX43 in astrocytes, which are important for maintaining neurogenesis, cause dysfunction. Some antidepressants, such as selective serotonin reuptake inhibitors (SSRIs), suppress microglial activation and promote adenosine triphosphate (ATP), brain‐derived neurotrophic factor (BDNF), and vascular endothelial growth factor (VEGF) release from astrocytes to restore neurogenesis. (c) Epilepsy is accompanied by increased neurogenesis and following depletion of neural stem cells (NSCs). Activated microglia phagocytose live newborn neurons, i.e., phagoptosis, and suppress the emergence of ectopic granule cells. Microglial TLR9 signaling also suppresses aberrant neurogenesis via TNF‐α release, whereas CX3CR1 and P2Y12 promote neurogenesis

Several studies have reported that impaired adult neurogenesis is associated with cognitive decline in AD (Figure 2A). Proliferation, maturation, and differentiation of abGCs were impaired in Tg2576 mice, which are a model of AD (Krezymon et al., 2013). In humans, the severity of neurofibrillary tangles and senile plaque accumulation are significantly correlated with a decreased number of hippocampal DCX‐positive immature neurons (Moreno‐Jiménez et al., 2019). Additionally, neurogenesis in both the SGZ and the SVZ has been shown to be impaired by Aβ oligomers and is considered an early event in AD neurodegeneration (Scopa et al., 2020).

Several studies have suggested that microglia are activated in the AD brain where they cause inflammation and alter their effects on neurogenesis. In a mouse model of AD, in which mutant human APP and mutant human presenilin‐1 (PS1) are expressed, treatment with minocycline improved the performance of hippocampal‐dependent learning tasks and increased survival of abGCs but did not restore dendritic shrinkage and spine loss (Biscaro et al., 2012). Moreover, depletion of microglia by treatment with colony stimulating factor 1 receptor‐inhibitors restored growth, differentiation, and survival of abGCs in AD model mouse expressing PS1 (Ortega‐Martinez et al., 2019).

Microglia phagocytose and remove dead neurons under physiological conditions, whereas activated microglia can phagocytose live neurons and execute cell death, a phenomenon triggered by inflammation and called as phagoptosis. For example, activated microglia perform phagoptosis of cerebellar neurons after exposure to LPS and Aβ (Fricker et al., 2012), and of abGCs after status epilepticus (SE; Luo et al., 2016). Thus, it is possible that phagoptosis of abGCs by microglia may contribute to the low survival rate of abGCs in AD. In addition, Choi et al. reported that co‐culturing primary microglia from mice expressing PS1 variants with wild‐type NPCs inhibited NPCs proliferation (Choi et al., 2008). These results suggest that activated microglia in AD impair multiple processes of neurogenesis.

Changes in astrocytic properties have also been reported in the AD brain. In the postmortem brain of human patients who had AD, astrocytes are activated in the areas around amyloid plaques in the SGZ, as determined by increased GFAP expression and enlarged cell bodies and processes (Kamphuis et al., 2014). In vitro studies demonstrated that exposure of astrocytes to Aβ induces the release of multiple cytokines including IL‐1β, IL‐6, and IL‐17, which leads to neuronal death (Garwood et al., 2011). Importantly, these cytokines have been shown to reduce neurogenesis in high concentrations (Liu et al., 2014; Valliéres et al., 2002; Wu et al., 2013; Zou & Crews, 2012). In the brains of APPswe/PS1dE9 AD mice, astrocytic expression of genes involved in differentiation and synaptic regulation of abGCs was decreased, suggesting that the ability of astrocytes to support neurogenesis was weakened (Orre et al., 2014). Indeed, neurons co‐cultured with astrocytes that were isolated from aged AD mice showed less neurite growth than those cultured with healthy astrocytes (Iram et al., 2016). In this research, astrocytes in aged AD mice have a relatively low expression of VEGF‐A, indicating a loss of function in astrocytes to support neurons. Therefore, it is possible that dysfunctions of astrocytic activities that support the growth of abGCs result in impaired neurogenesis in AD.

### 5.2 Depression

Depression is one of the most common mental diseases. Impaired neurogenesis has been implicated as an important cause of depression (Jacobs et al., 2000; Figure 2B). Various stresses such as unpredictable stress, restraint, unavoidable foot shock, social defeat have been shown to induce depressive behaviors and decreased neurogenesis in animals (Heine et al., 2005; Jayatissa et al., 2006; Khawaja et al., 2004; Vollmayr et al., 2003). It has been also shown that enhanced neurogenesis is sufficient to improve anxiety and depression‐related behaviors caused by chronic corticosterone administration (Hill et al., 2015). Treatment with antidepressant drugs such as serotonin selective reuptake inhibitors (SSRIs) and serotonin noradrenaline reuptake inhibitors were shown to promote neurogenesis (Malberg et al., 2000; Xu et al., 2006). Many antidepressants used to treat depression in humans alter monoamine levels within hours, but effective changes in mood can be observed after 3–4 weeks (Machado‐Vieira et al., 2010); this time period coincides with the time window needed for abGCs to mature (Aimone et al., 2014). Together, these data provide support for the current consideration of neurogenesis as a therapeutic target of depression.

Appel et al. (2018) revealed that mice with microglial dysfunction exhibit abnormal neurogenesis and depression‐like behaviors (Appel et al., 2018). Mice in which vacuolar sorting protein 35, which is essential for selective endosome‐to‐Golgi retrieval of membrane proteins, is knocked out showed elevated microglia density in the hippocampus as well as abnormal microglial morphology. In addition to exhibiting depression‐like behaviors, abGCs in these mice exhibited a decreased number and malformation of dendrites of abGCs in addition to malformed dendrites of abGCs (Appel et al., 2018). These results suggest that alterations in microglial function contribute to the disruption of neurogenesis and depression‐like behavior. Few studies have investigated the direct effects of microglial regulation of neurogenesis on depression and a further investigation is necessary.

In contrast with the microglial activation and resulting inflammation that is observed in patients with depressive disorders, the number of astrocytes has been shown to be decreased in the amygdala, dorsolateral prefrontal cortex, and hippocampus of these patients (Bowley et al., 2002; Cobb et al., 2016; Cotter et al., 2002). Therefore, it is hypothesized that the decrease in astrocytic support of neurogenesis underlies the pathogenesis of depression; it has been suggested that recovery or enhancement of astrocytic neurogenesis support is useful for the treatment of depression. Administration of an SSRI increased hippocampal BDNF levels and promoted neurogenesis (Terada et al., 2014). Treatment with an SSRI increased the expression of BDNF in astrocytes by promoting ATP release via the vesicular nucleotide transporter and presumably activating the ATP receptor in an autocrine manner (Kinoshita et al., 2018). In the same study, the inhibition of ATP release from astrocytes quenched both the effect of the SSRI and the production of BDNF. Although not validated in this study, ATP can also act directly on NPCs to promote proliferation (Cao et al., 2013). Additionally, overexpression of BDNF in astrocytes increased neurogenesis (Quesseveur et al., 2013). These results suggest the possibility that SSRI treatment elicits an anti‐depressive effect by increasing BDNF expression in astrocytes and consequently promoting neurogenesis. Antidepressants also increase the secretion of GDNF secretion in vitro from rat primary astrocytes and the glia‐like cell line C6 (Hisaoka et al., 2001). GDNF acts on NPCs and promotes their differentiation to astrocytes in vitro. The promotion of NPC differentiation to astrocytes may indirectly support and enhance neurogenesis.

It has been suggested that the reduced expression of genes associated with astrocytic function is also associated with reduced neurogenesis in depression. Rats exposed to chronic unpredictable stress exhibited depression‐like behaviors; the collapse of gap junctions between astrocytes was observed in these rats, which was associated with reduced expression of astrocytic connexin 43 (CX43; Sun et al., 2012). CX43 forms gap junctions which are an essential component of intercellular communication. Proliferation of abGCs were inhibited in mice with astrocyte and radial glia cell‐specific knockout of CX43 (Zhang et al., 2018).

The expression of aquaporin‐4 (AQP4), a principle water channels in the brain that is expressed mainly by astrocytes, was reduced in patients with depression (Bernard et al., 2011). Since AQP4 knockout suppresses neurogenesis, the function of this water channel is likely important for maintaining neurogenesis (Kong et al., 2008). In addition, APQ4 knockout mice showed depression‐like behaviors after chronic injection of corticosterone (Kong et al., 2014). These results suggest that AQP4 may control the development of depression by regulating astrocyte function and adult neurogenesis.

Though most antidepressant drugs have been designed with a focus on neurons, a multifaceted approach that targets multiple cells, including microglia and astrocytes, may provide a breakthrough for the treatment of depressive patients with drug resistance.

### 5.3 Epilepsy

Epilepsy is one of the most common neurological diseases in younger populations. It is characterized by excessive hypersynchronous discharge‐induced seizures (Stafstrom & Carmant, 2015; Figure 2C). In animal models of temporal lobe epilepsy, neurogenesis characterized by a rapid increase in proliferation over the weeks after SE and eventual depletion of NSCs. (Jessberger et al., 2005; Luo et al., 2016; Sierra et al., 2015). abGCs in the epileptic brain exhibit abnormal structural features such as basal dendrites extending to the hilus, mossy fiber sprouting, and ectopic migration to the hilus (Jessberger et al., 2007). These abnormal abGCs are thought to contribute to the exacerbation of epilepsy. Hilar ectopic abGCs receive much more excitatory input than abGCs of the same age that are normally integrated in the granule cell layer (Althaus et al., 2019). There is a significant correlation between the number of ectopic granule cells and seizure frequency (Hester & Danzer, 2013). Genetic removal of abGCs after SE significantly reduced both the number of ectopic granule cells and the frequency of spontaneous recurrent seizures (Cho et al., 2015). In addition, pharmacological suppression of abGC activity dramatically reduced spontaneous seizures (Zhou, et al., 2019) and sustained inhibition of neurogenesis after SE induced a transient, though incomplete, suppression of spontaneous seizures (Varma et al., 2019). These results suggest that abGC produced rapidly after SE contributes to the development of epilepsy.

Suppression of neurogenesis during the chronic phase of epilepsy may not affect the exacerbation of epileptic seizures but may be related to multiple symptoms associated with epilepsy. For example, patients with epilepsy have a higher risk of depression than the general population (Tellez‐Zenteno et al., 2007). Depression‐like behaviors were observed in rats after pilocarpine‐induced SE (Mazarati et al., 2009) and decreased neurogenesis can be a cause of depression (Jacobs et al., 2000). Thus, it is possible that attenuated neurogenesis during the chronic phase of epilepsy may be associated with the onset of depression. abGC is also involved in processes such as spatial cognition and pattern separation (Anacker & Hen, 2017), which may cause cognitive impairment in epilepsy (Holmes, 2015). Therefore, the control of both the number and differentiation of abGCs after SE may be a therapeutic target to prevent the progression of epilepsy.

Microglia have been shown to modulate neurogenesis in the epileptic brain. Microglia after kainate‐induced SE have been shown to suppress neurogenesis via TLR9‐dependent TNF‐α secretion, and to reduce the number of ectopic granule cells (Matsuda et al., 2015). Activated microglia also maintain homeostasis of neurogenesis by phagocytosing excess abGCs. In the healthy brain, microglia mainly engulf apoptotic abGCs, but activated microglia after SE mainly engulf cleaved‐caspase3‐negative live abGCs (Luo et al., 2016). The authors have shown that minocycline treatment suppressed the activation of microglia, reduced the number of engulfed abGCs, and increased the number of ectopic granule cells. These results suggest that microglia suppress excess proliferation of NSCs and eliminate abGCs after SE to inhibit the formation of abnormal neural circuits that lead to the disruption of synaptic E/I balance. However, in contrast with these results, some studies have shown that microglia accelerate neurogenesis after SE. In the rat lithium pilocarpine model, suppression of microglial activation with minocycline reduced the number of ectopic granule cells 14 days after SE (Yang et al., 2010). In addition, administration of a function blocking CX3CR1 antibody to the hippocampus after SE suppressed microglial activation and decreased immature neurons (Ali et al., 2015). Similar results were obtained in P2Y12R knockout mice, in which microglia did not extend their processes toward neurons after SE in response to ATP released from neurons (Mo et al., 2019). These discrepancies regarding the role of microglia in neurogenesis after SE may be due to differences in experimental conditions. There are various methods for creating mouse and rat models of epilepsy, such as the pilocarpine model, the kainate model, and the kindling model. In addition, there are multiple routes of administration including intraperitoneal, intrahippocampal, intraventricular, and intrastriatal, which may induce different effects on glial activation. Indeed, microglia show different gene expression in the kainite intrahippocampal model and the pilocarpine intraperitoneal model (Benson et al., 2015). Thus, we need to carefully assess the results from reports using different models of epilepsy.

In epileptic patients, especially those with hippocampal sclerosis (a characteristic structural abnormality in the epileptic hippocampus associated with anti‐epileptic drug resistance), astrocytes are both activated and actively proliferating (Thom, 2014). Also in these patients, astrocytes in the dentate gyrus form gliosis and the length of astrocytic fibers surrounding the granule cell layer significantly increases (Thom, 2014). Although the dysfunction of astrocytes in epilepsy is well‐known, surprisingly little is known about whether and how astrocytes regulate neurogenesis in the epileptic brain. One study showed that basal dendrites arising from abGCs after SE were often in contact with astrocytes (Shapiro et al., 2005). Inhibition of astrocyte metabolism by administration of fluorocitrate to the dentate gyrus failed to prevent basal dendritic formation (Shapiro et al., 2005). After KA‐induced severe seizures, the expression of nestin was increased in astrocytes, suggesting that the release of factors related to neuronal migration and location may be promoted (Yang et al., 2008). In addition, the expression of Netrin 1 and Sema‐3A, proteins that regulate neurite formation and cell migration, were increased in the hilus (Yang et al., 2008). It is not clear whether astrocytes can secrete Netrin 1, but they can secrete Sema‐3A (Molofsky et al., 2014) and may be involved in the appearance of ectopic granule cells.

Astrocytes in epileptic patients also have a variety of dysfunctions, including reduced expression of the inwardly rectifying potassium channel subtype Kir4.1, which is responsible for the potassium buffering action of astrocytes; impaired AQP4 function; and disruption of the homeostasis properties of glutamate and gamma‐amino butyric acid (GABA; Kong et al., 2008). Knockout of AQP4 is known to impair the proliferation, differentiation, and migration of SVZ NPCs (Kong et al., 2008). Glutamate and GABA are neurotransmitters that can directly control neurogenesis; disruption of their homeostasis may trigger abnormal neurogenesis (Nakamichi et al., 2009).

Compared to these studies which investigated the role of microglia and astrocytes in the acute increase in neurogenesis after SE, their role in the depletion of NSCs in the chronic phase has been poorly investigated. A study by Sierra et al. (2015) indicates that depletion of NSCs is due to an enhanced differentiation of NSCs to reactive astrocytes (Sierra et al., 2015). Because the differentiation of NSCs to neurogenesis can be shifted to astrogliogenesis by IL‐6 released from microglia and astrocytes (Monje et al., 2003; Nakanishi et al., 2007), IL‐6 is a possible candidate that induce the suppression of neurogenesis in chronic epilepsy. However, LPS‐induced inflammation alone did not result in similar NSC depletion (Sierra et al., 2015). The involvement of microglia and astrocytes in altering the fate of NSCs needs to be further studied.

In summary, an increasing number of studies have suggested the involvement of microglia and astrocytes in abnormal neurogenesis in the epileptic hippocampus, but some findings are contradictory, likely due in part to differences in experimental conditions.

## 6 CONCLUSIONS

The hippocampal SGZ is one of the limited areas in which neurons are continuously produced throughout life. Adult neurogenesis is modulated by the environment in the neurogenic niche, and microglia and astrocytes are key players in controlling neurogenesis. Microglia support neurogenesis through phagocytic removal of unnecessary structures to create space for the incorporation of abGCs into pre‐existing neuronal circuits, changes in synaptic plasticity upon contact, and the release of growth factors. Astrocytes also release growth factors and various soluble factors to stimulate neurogenesis. It is not clear, however, whether microglia and astrocytes in the SGZ have specially tailored profiles, i.e., neurogenesis‐related heterogeneity, to support neurogenesis.

Interestingly, the reduced or abnormal neurogenesis that occurs in aging and neurodegenerative diseases may result from dysfunction of these glial cells. Experiments using genetically modified animals and treatment with therapeutic agents indicate that the improvement of some symptoms can be achieved through restoration of glial cell function and normalization of neurogenesis, suggesting that the modulation of glial function for the purpose of manipulating neurogenesis can provide a source of novel therapeutic targets. However, the ways in which these glial functions that modulate neurogenesis differ between each disease should be clarified. In addition, it is necessary to capture not only changes in individual cell types but also changes in the neurogenesis niche as a whole.

## CONFLICT OF INTERESTS

The authors have no conflicts of interest to report.

## AUTHOR CONTRIBUTIONS

T.A. and R.K. wrote the manuscript. T.A., R.K., and Y.I. discussed and commented on the manuscript.

### Peer Review

The peer review history for this article is available at https://publons.com/publon/10.1111/ejn.14969.

## Data Availability

This is a review article and does not include experimental data.
